# Unraveling the complex role of tumor-associated neutrophils within solid tumors

**DOI:** 10.1007/s00262-025-04049-5

**Published:** 2025-05-19

**Authors:** Yingxin Wang, Jiakang Ma, Yuhao Liu, Weiheng Cui, Xiaodong Chu, Yusheng Lin, Lu Wang

**Affiliations:** 1 Institute of Precision Cancer Medicine and Pathology, School of Medicine, and Minister of Education Key Laboratory of Tumor Molecular Biology, and State Key Laboratory of Bioactive Molecules and Druggability Assessment, Jinan University, 601 Huangpu Avenue West, Guangzhou, 510632 Guangdong China; 2 Department of General Surgery, The First Affiliated Hospital of Jinan University, Guangzhou, China; 3 Department of Thoracic Surgery, The First Affiliated Hospital of Jinan University, Guangzhou, China

**Keywords:** Neutrophils, Hepatocarcinoma, Breast cancer, Gastric cancer, Colorectal cancer, Pathways

## Abstract

Neutrophils are integral to the frontline defense against pathogenic bacterial and fungal invasions. Beyond their traditional roles, these cells are increasingly recognized for their dualistic contributions to the pathology of autoimmune and inflammatory diseases, as well as their complex involvement in cancer progression. Neutrophils interact with different disease states, highlighting their potential as therapeutic targets. Within tumor microenvironment (TME), tumor-associated neutrophils (TANs) exhibit a functional dichotomy, capable of either fostering or impeding tumor growth and metastasis. This binary functional potential of TANs, under certain conditions, suggests a reversible state that could transition from tumor-promoting to tumor-eradicating phenotypes. Despite the critical implications of such functional plasticity, systematic studies of TAN behavioral shifts in the context of cancer immunotherapy remain scarce. Herein, we review recent advancements in the understanding of TANs within the TME, highlighting their binary regulatory effects on solid tumors. Leveraging the latest insights from experimental and clinical research, this review elucidates the complex roles of TANs in tumor development and explores their molecular interactions as potential therapeutic targets. The elucidation of these mechanisms holds promise for novel cancer treatment strategies, aiming to improve patient outcomes by manipulating the tumor-promoting or -suppressing functions of TANs.

## Background

Neutrophils, which arise from hematopoietic stem cells in the bone marrow, have been traditionally acknowledged in immunology for their phagocytic activity and bactericidal capabilities, as well as their role in orchestrating inflammatory responses [1]. Recent research reveals a more complex role for these cells within the tumor milieu. Neutrophil infiltrates in tumors, known as TANs, have emerged as pivotal contributors to cancer progression. TANs facilitate processes such as angiogenesis, enhance the migration of neoplastic cells, and modulate the immune landscape, effectively acting as an immunosuppressive switch that orchestrates the behavior of various immune cell populations [2].

Cancer development results from a complex interplay of genetic predispositions and environmental factors, with certain malignancies linked to harmful lifestyle factors [3]. Within the TME, cancer cells expertly manipulate TANs via a milieu of secreted cytokines, chemokines, and other effector molecules [4]. This interaction propels TANs toward a dichotomous polarization, adopting either an anti-tumoral (N1) or a pro-tumoral (N2) phenotype. N1 TANs are characterized by an upsurge in pro-inflammatory mediators such as tumor necrosis factor-α (TNF-α) and C–C motif chemokine ligand 3 (CCL3), along with enhanced expression of intercellular adhesion molecule-1 (ICAM-1) and reduced arginase activity. CCL3 promotes inflammation and immune responses within the TME [5]. In contrast, N2 TANs exhibit overexpression of chemokines such as CCL2, CCL4, CCL8, CCL12, and CCL17 [6]. Importantly, while CCL3 is involved in pro-inflammatory responses in the N1 phenotype, its role can vary in different contexts, such as contributing to fibrosis in the liver, highlighting the importance of considering specific tumor types and microenvironments.

The roles of neutrophils are shaped by their surrounding environment, influencing their functions in cancer biology. Recent studies have identified mechanisms that affect TAN polarization. For example, in non-small cell lung carcinoma, Smad3 has been recognized as crucial for TAN polarization, especially via TGF-β/Smad3 axis. In experimental models, TANs transition from a predominantly N2 state in wild-type mice to an N1 state in Smad3-knockout (KO) mice, correlating with enhanced neutrophil infiltration and tumor regression. Single-cell analysis reveals that a subset of TANs in the Smad3-KO TME exhibits a mature N1 phenotype, while wild-type TANs remain in an immature N2 state due to Smad3 presence [7]. Another study found that acrolein produced by glioma cells under hypoxia inhibits neutrophil activation and induces an anti-inflammatory phenotype by interacting with Cys310 of protein kinase B (AKT), thereby inhibiting AKT activity [8]. Additionally, research on a dendritic cell-based hepatocellular carcinoma neoantigen nano-vaccine showed that acidic-triggered captopril release can repolarize pro-tumoral N2 neutrophils to an anti-tumoral N1 phenotype [9]. Moreover, melanoma-derived extracellular vesicles (EVs) may activate neutrophils, promoting their migration toward the TME and supporting N2 polarization, thus contributing to tumor progression [10].

The interaction between neutrophils and tumor cells is imperative in regulating tumor growth and progression within the TME. TANs secrete a variety of cytokines, including transforming growth factor β2 (TGF-β2), IL-1β, TNF-α, IL-12, granulocyte colony-stimulating factor (G-CSF), vascular endothelial growth factor (VEGF), CC family chemokines, and CXC family chemokines, etc. [11]. The release of these cytokines promotes angiogenesis, suppresses anti-tumor immune responses, and creates a supportive environment for tumor cells, facilitating growth and metastasis. During tumor development, each tumor cell undergoes metabolic reprogramming to cope with challenges like hypoxia and nutrient deficiency. In this context, tumor cells can induce an oxidative phenotype in neutrophils through the stem cell factor (SCF) signaling pathway, causing DNA damage in neighboring cells, promoting inflammation, and enhancing tumor cell survival and proliferation [12].

Despite increasing interest in targeting neutrophils for cancer therapy, the molecular details of neutrophil-tumor interactions remain unclear. Understanding these pathways is essential for advancing precision oncology and developing targeted therapies. This review summarizes current insights into the molecular mechanisms by which TANs influence liver, breast, gastric, and colorectal cancers, and highlights the potential of TANs as a focal point for innovative cancer treatment strategies.

## Biological characterization and functionality of neutrophils

**Fig. 1:**
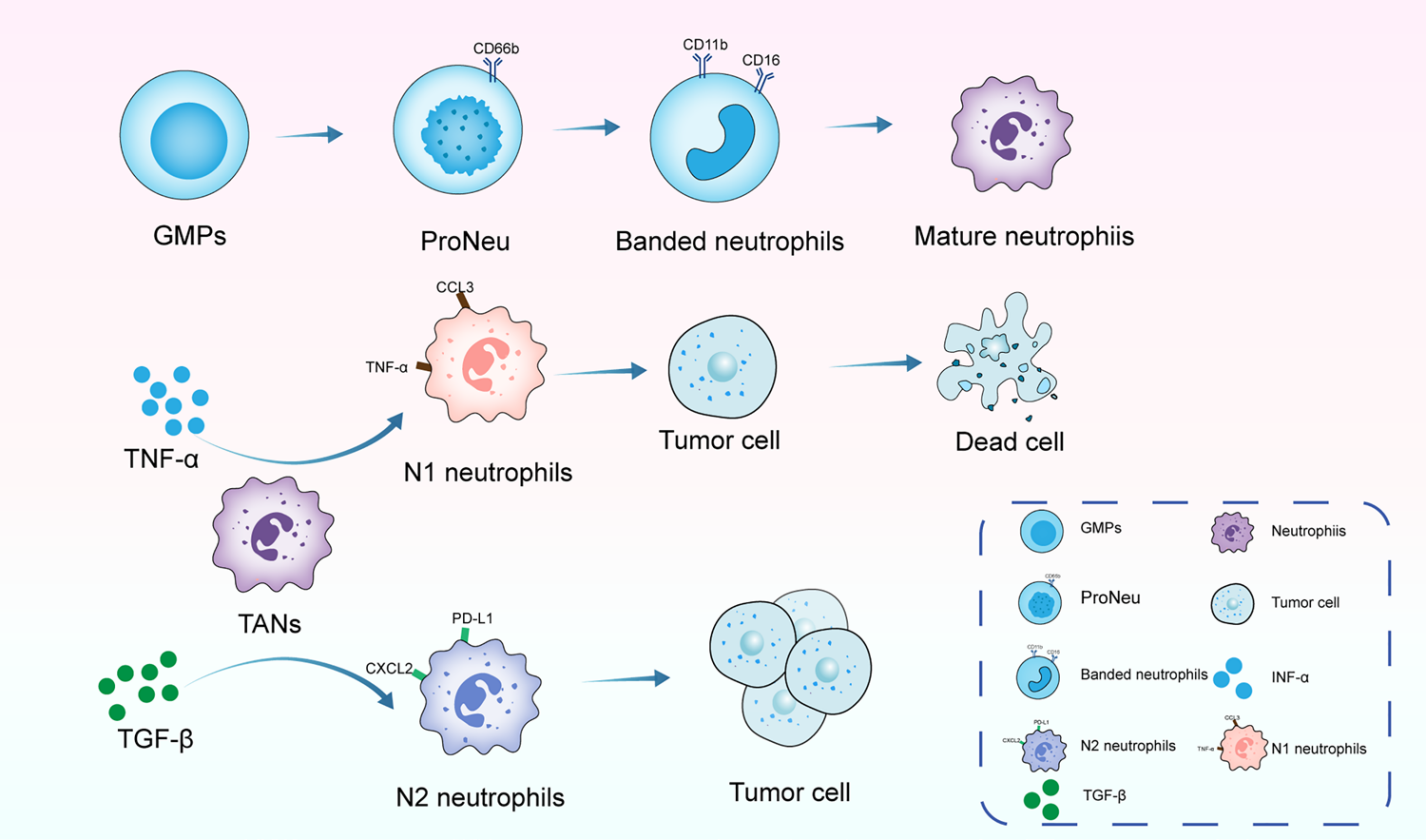
The differentiation trajectory of neutrophils and their polarization within the tumor microenvironment. This trajectory encompasses various stages of development. In the tumor microenvironment, TGF-β promotes the polarization of tumor-associated neutrophils (TANs) into a pro-tumoral N2 phenotype. In contrast, the release of TNF-α facilitates the polarization of TANs toward an anti-tumoral N1 phenotype

Neutrophils, the predominant class of myeloid lineage cells in human circulation, originate and mature within the bone marrow from hematopoietic progenitors. Their differentiation involves a series of stages where common myeloid progenitors branch into multipotent granulocyte-monocyte progenitors (GMPs). These GMPs then give rise to precursors of neutrophils and monocytes (Fig. 1). A key stage in neutrophil maturation is the expression of CD66b on promyelocytes, followed by increased CD11b and CD16 expression, leading to the formation of band and segmented neutrophils [13]. Within the TME, these cells exhibit remarkable heterogeneity and plasticity. Studies indicate that TGF-β steers TANs toward the N2 phenotype, while its inhibition promotes N1 TANs [14].

In solid tumors, neutrophils express specific markers. Immunohistochemical analysis revealed that CCL2^+^ and CCL17^+^ cells, which also express the neutrophil marker CD66b, are found throughout hepatocellular carcinoma stroma [15]. CD66b^+^ TANs and CD8^+^ tumor-infiltrating lymphocytes (TILs) have prognostic significance in the invasive margins of colorectal cancer (CRC). Additionally, the infiltration of CD163^+^, CD68^+^, and CD66b^+^ cells in gastric cancer tissue was significantly increased and independently associated with gastric cancer prognosis [16].

Neutrophils are classically known for their roles in executing bacterial phagocytosis and mediating inflammatory processes. Their strategies against pathogens include phagocytosis, reactive oxygen species (ROS) generation, protease secretion, and forming neutrophil extracellular traps (NETs). NETs, which consist of DNA, histones, and enzymes, are used by activated neutrophils to capture and eliminate pathogens [17]. Furthermore, neutrophils aid adaptive immunity by presenting antigens through major histocompatibility complex (MHC) class I, facilitating the maturation of naïve CD8^+^ T cells into cytolytic T cells [18].

The inherent diversity and plasticity of neutrophils underpin the dual potential exhibited by TANs within the TME [13]. N1 TANs can kill cancer cells by releasing ROS and reactive nitrogen species (RNS) and can activate T cells and recruit pro-inflammatory (M1) macrophages. Conversely, N2 TANs promote oncogenesis by releasing matrix metalloproteinase 9 (MMP9), which aids angiogenesis and tumor cell spread, while inhibiting natural killer (NK) cell function. They can also recruit anti-inflammatory (M2) macrophages and regulatory T cells [19].

**Fig. 2:**
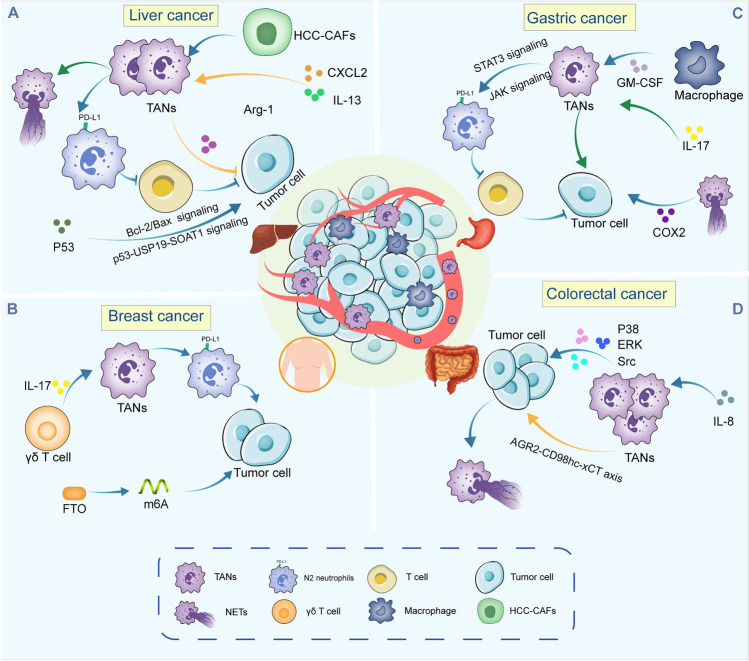
A comprehensive overview of the regulatory mechanisms governing tumor-associated neutrophils (TANs) in solid tumors. The mechanisms regulating TANs are illustrated in the contexts of breast cancer (BC), colorectal cancer (CRC), gastric cancer (GC), and hepatocellular carcinoma (HCC)

Neutrophils exhibit a dual role in tumor development. N1 TANs directly kill tumor cells by releasing cellular hydrolases and producing ROS and myeloperoxidase (MPO) through degranulation. They can produce immune factors like TNF-α, activating immune cells such as natural killer cells (NKTs), dendritic cells (DCs), and macrophages [20]. A study discovered that N1 TANs can activate peripheral blood CD4^+^ T cells by binding their Fc receptors to IgG Fc receptor proteins on tumor cells, leading to tumor cell destruction and reduced tumor growth [21]. On the other hand, N2 TANs foster cancer progression via releasing ROS, cytokines, neutrophil elastase (NE), and NETs, among other factors [22] (Fig. 2).

## Molecular mechanisms of TANs in solid tumor development and progression

TANs stimulate angiogenesis, generate NETs, and modulate the immune system to support the growth and dissemination of malignancies. Recent studies highlight the tumor-promoting effects of TANs in high-incidence solid tumors, including liver cancer, breast cancer, CRC, and gastric cancer. This section summarizes the molecular mechanisms by which TANs promote cancer progression in solid tumors (Table 1).

**Table 1:** Functions of TANs in different tumors

Cancer type	Related factor	Mechanism and function	References
Hepatocellular carcinoma	IL-6	Hepatocellular carcinoma-CAFs induce TANs differentiation into PDL1^+^ neutrophils through the IL6-STAT3-PDL1 signaling cascade to inhibit T cells	[25]
	CXCL1/CXCL2/CXCL5	Recruit neutrophils and inhibit T cells	[6]
	P53	promote the hepatocellular carcinoma progression via BCL-2/BAX axis and P53-USP19-SOAP1 axis	[75]
	MMP9	Stimulate angiogenesis activity in tumor cells	[73]
	BMP2, TGF-β2	Increase stem cell characteristics in tumor cells	[73]
	CCL2/CCL17	Recruit macrophages and Tregs, promote tumor progression	[30]
Breast cancer	IL-17A	Promote N2-like neutrophil polarization	[57]
	FTO	Regulate EMT and promote breast cancer	[58]
	Oncostatin M	Induce the expression of VEGF	[76]
	β2-integrin	Bind to ICAM-1 of tumor cells and promote cell migration	[76]
	LTs	Selectively expand tumor cells with high tumorigenic potential and aid their colonization	[76]
Gastric cancer	GM-CSF	Induce PD-L1 expression on neutrophils	[76]
	TGF-β1	Activate neutrophils and promote tumor cell EMT	[63]
	IL-6	Promote the migration of gastric cancer cells and the tube formation of endothelial cells	[77]
	COX-2	Enhance the metastatic ability of gastric cancer cells	[65]
	IL-17A	Activate JAK2/STAT3 signaling pathway and promote EMT of tumor cells	[62]
Colorectal cancer	IL-8	Induce the activation of neutrophils and NET formation	[69]
	CXCL2	Promote colon cancer cells adhesion and growth in the peritoneal wall	[70]
	CEACAM1	Stimulate the relocation of CRC cells to the liver	[71]
	AGR2	Promote migration of tumor cells	[11]
Prostate cancer	STAT5	Neutrophil-mediated PCa killing was found to be mediated by suppression of STAT5	[102]

*BMP* bone morphogenetic protein, *FTO* fat mass and obesity-associated protein, *LT* lymphotoxin, *CEACAM* carcinoembryonic antigen-related cell adhesion molecule

### The multifaceted role of neutrophils in hepatocellular carcinoma

Neutrophil proliferation in the TME accelerates tumor angiogenesis, epithelial-mesenchymal transition (EMT), and growth by producing MMP9, NETs, and hepatocyte growth factor, worsening hepatocellular carcinoma and its metastasis [23]. Here, we summarize the critical molecular mechanisms involved in the development of hepatocellular carcinoma.

#### Immunomodulatory actions of neutrophils via the PD-1/PD-L1 pathway

Programmed death ligand 1 (PD-L1) is a glycoprotein that binds to programmed death receptor 1 (PD-1) [24]. A recent study showed that cancer-associated fibroblasts (CAFs) induce the differentiation of TANs into PD-L1^+^ neutrophils through the IL-6/STAT3/PD-L1 pathway. Co-culturing purified peripheral CD3^+^ T cells with HCC-CAF-primed neutrophils significantly reduced T-cell IFNγ production and proliferation (by 40%). Adding PD-L1 neutralizing antibodies to these cultures attenuated T-cell suppression [25]. Notably, neutrophils primarily infiltrate peritumoral tissues in hepatocellular carcinoma patients, where the neutrophil-to-lymphocyte ratio (NLR) is elevated compared to intratumoral tissues, and high levels of PD-L1 expression are observed. Researchers further confirmed a greater prevalence of PD-L1^+^ neutrophils and PD-1^+^ T cells in hepatoma-bearing mice, indicating that neutrophils mediate tumor immune escape by upregulating PD-L1 expression [26]. Some studies suggest that tumor cells may reprogram CD10^+^ALPL^+^ neutrophils, leading to the “irreversible” exhaustion of T cells [27].

Clinically, safe and effective agents that inhibit STAT3 activation may offer avenues for hepatocellular carcinoma prevention and treatment. For instance, α-mangostin (α-MGT) was found to inhibit STAT3 activation and exert anti-hepatocellular carcinoma effects, significantly suppressing cell proliferation and inducing apoptosis in various cancer cell lines, including HepG2 and SK-Hep-1. Moreover, α-MGT inhibited the expression of STAT3-regulated genes [28], suggesting its potential as a therapeutic agent. Other compounds, like emodin and evodiamine, have also shown promise in inhibiting STAT3 signaling.

#### The chemokine axis and neutrophil mobilization: exploring the CXCL paradigm in hepatocellular carcinoma

C-X-C motif chemokine ligands (CXCLs) are small cytokines that recruit neutrophils through CXCL5, CXCL2, and CXCL1, modulating T-cell responses. Neutrophil recruitment is influenced by monocytes, macrophages, or epithelial cells [28]. CXCL2 and IL-13 play crucial roles in promoting neutrophil recruitment and upregulating arginase-1 (Arg1), which suppresses T-cell activation and aids tumor progression [29]. CXCL5 binds primarily to the chemokine receptor CXCR2. Although the precise activation mechanisms via CXCR2 are not fully understood, CXCL5 is regulated by TGF-β and Axl signaling, facilitating neutrophil infiltration in HCC patients. Additionally, CXCL5 acts as an effector of TANs, promoting macrophage and regulatory T-cell infiltration through other chemokines like CCL2 and CCL17 [30]. Studies have shown that active serine proteases can downregulate CXCL2 through targeted cleavage, reducing neutrophil recruitment and suppressing hepatocellular carcinoma progression [31]. Moreover, brahma-related gene 1 (Brg1) expression levels demonstrate a positive correlation with CXCL14 and neutrophil infiltration [7]. Recent research indicates that inhibiting CXCR2 can reprogram the TME, transitioning TANs from an N2 to an N1 phenotype [32].

#### The role of NETs in tumor formation in hepatocellular carcinoma

NETs are extensive, web-like structures released extracellularly and composed of cytosolic, granular, and nuclear proteins. These structures play a significant role in promoting cancer metastasis and immune evasion. In a recent study, researchers demonstrated that neutrophils promote hepatocellular carcinoma metastasis through the enhanced formation of NETs [33], which trigger a tumorous inflammatory response via the activation of the Toll-like receptors 4/9-cyclooxygenase 2 (TLR4/9-COX-2) axis, thereby fueling metastasis [34]. Moreover, in investigations related to non-alcoholic steatohepatitis (NASH)—a condition that can promote liver cancer—researchers found that the inhibition of NET formation, either through treatment with deoxyribonuclease (DNase) or using peptidyl arginine deaminase type IV knockout mice (PAD4–/–), did not inhibit the development of fatty liver but altered the pattern of liver inflammation, ultimately resulting in reduced tumor growth [35]. In a controlled study involving hepatitis B virus (HBV)—infected individuals, researchers observed elevated levels of NETs in serum and tissue specimens from hepatocellular carcinoma patients infected with HBV and concluded that the HBV-mediated S100A9-TLR4/RAGE-ROS cascade facilitates the growth and metastasis of hepatocellular carcinoma [36]. Furthermore, recent clinical studies have indicated that detecting NET formation in tumors can effectively predict prognosis and response to immunotherapy in hepatocellular carcinoma patients.

#### Delving into the P53 tumor suppression mechanism in hepatocellular carcinoma

P53 serves as a critical tumor suppressor, functioning as a transcription factor that regulates DNA repair and induces cell cycle arrest in response to oncogenic stress. Mouse model studies show that significant decreases or chronic hyper-activation of p53 can lead to cancer initiation [37]. p53 inhibits tumor growth by regulating CDC20, essential for cell cycle progression from G2 to M phase. It negatively regulates CDC20 expression by binding its promoter region, preventing premature cell cycle progression. In contrast, mutant p53 fails to regulate CDC20, leading to increased CDC20 levels and uncontrolled cell division. Flow cytometry analyses indicate that CDC20 suppression results in significant G2/M phase cell cycle arrest in HCC cells. High CDC20 expression allows cells with damaged DNA to escape mitosis and evade apoptosis, facilitating hepatocellular carcinoma development [38]. Conversely, the absence of p53 can increase cholesterol esterification through the p53-USP19-SOAT1 signaling axis, thereby facilitating hepatocarcinogenesis [39]. Reticulons (RTNs) facilitate p53 Ser392 phosphorylation via Chk2, inhibiting hepatocellular carcinoma growth and inducing apoptosis [40]. Another study found that ZNF498, a p53 Ser46 phosphorylation suppressor, is highly expressed in hepatocellular carcinoma and correlates positively with advanced disease and poor survival [41]. Moreover, FAT10 has been suggested to promote hepatocellular carcinoma development by mediating p53 degradation [42]. Collectively, p53 is involved in multiple liver cancer development stages, and targeting p53 may provide a novel therapeutic approach.

#### Navigating the PI3K/Akt/mTOR signaling pathway in hepatocellular carcinoma

The PI3K/Akt/mTOR pathway is crucial for regulating the cell cycle, proliferation, apoptosis, and metabolism, and is activated in many cancers due to dysregulated receptor tyrosine kinases (RTK) [43]. The fatty acid receptor CD36 is implicated in both lipid and glucose metabolism in the liver. The overexpression of CD36 leads to increased glycolytic flux and lactic acid production via activation of the Src/PI3K/AKT signaling axis, which promotes hepatocellular carcinoma growth and metastasis [44]. Additionally, SOS Ras/Rac Guanine Nucleotide Exchange Factor 1 (SOS1) may induce EMT through the PI3K/Akt/mTOR pathway, thereby enhancing the invasion, migration, and metastasis of hepatocellular carcinoma cells [45]. In past studies, researchers found that elevated phosphoinositide-dependent protein kinase 1 (PDK1) expression, valosin-containing protein (VCP) interaction with high mobility group box-1 **(**HMGB1), and suppressor of cytokine signaling 5 (SOCS5) inhibition can promote hepatocellular carcinoma progression through the PI3K/AKT/mTOR pathway [46]. Long non-coding RNA (lncRNA) has been a hot topic recently. Some studies indicated that 67 dysregulated lncRNAs associated with the PI3K/AKT/mTOR pathway show oncogenic or anti-oncogenic effects in hepatocellular carcinoma by regulating epigenetic, transcriptional, and post-transcriptional levels [47]. Circular RNAs (circ RNAs) are also crucial ncRNAs that are sensitive to the tumor immune response. hsa_circ_0001727 (circZKSCAN1) has been reported to be a tumor-associated circRNA by sponging microRNAs. CircZKSCAN1 encodes circZKSaa, which sensitizes hepatocellular carcinoma cells to sorafenib via ubiquitination of mTOR. Overexpression of circZKSaa inhibits the development of hepatocellular carcinoma through the PI3K/Akt/mTOR pathway [48].

The PI3K/Akt/mTOR pathway is a promising target for anti-cancer compounds. Various dietary compounds, especially flavonoids, have been shown to inhibit this pathway in different cancers [49]. For instance, LY3023414 is a PI3K-AKT-mTOR inhibitor that suppresses human skin squamous cell carcinoma growth both in vitro and in vivo. Moreover, some researchers found that deoxyshikonin, isolated from Arnebia euchroma, inhibited CRC through the PI3K/Akt/mTOR pathway [50]. PI3K serves as a pivotal initiator, contributing to various malignant biological processes, including proliferation, apoptosis, and angiogenesis. Many PI3K inhibitors (PI3Ki) have been validated or advanced into clinical trials as antineoplastic agents. One study revealed that DZW-310 (a novel PI3Ki) significantly diminished hepatocellular carcinoma cell growth by promoting intrinsic apoptosis and G0/G1 phase cell arrest. Furthermore, DZW-310 suppressed angiogenesis by regulating the hypoxia-inducible factor (HIF)-1α/VEGFA axis. DZW-310 shows promise as a therapeutic agent for hepatocellular carcinoma and may expand the clinical applications of PI3K inhibitors in treatment [24].

### The role of neutrophils in the metastasis and advancement of breast cancer

Breast cancer is among the three most common cancers worldwide and is a leading cause of death in post-menopausal women, accounting for 23% of all cancer-related fatalities. While only 5–10% of breast cancer cases are linked to genetic factors, 90–95% are influenced by environmental and lifestyle factors, such as ionizing radiation, hormonal therapy, reproductive behaviors (like later childbirth), alcohol consumption, dietary habits, obesity, and physical inactivity [51]. Additionally, the neutrophil-to-lymphocyte ratio (NLR) has been identified as a prognostic marker for poor outcomes in breast cancer. Ordered multinomial logistic regression analysis demonstrated a positive correlation between NLR, carcinoembryonic antigen (CEA), and CA15-3 with tumor-node-metastasis (TNM) staging, based on data from 653 breast cancer patients and 100 patients with breast fibroadenoma [52]. Neutrophils impact breast cancer progression through several complex mechanisms, including the formation of NETs [53].

Mechanistically, NETs enhance the interaction between nuclear factor kappa B (NF-κB) essential modifier (NEMO) and IκB kinase (IKK)α/β, promoting NF-κB activation. One study suggests that NF-κB associates with NETs to establish a positive feedback loop that facilitates breast tumor progression and metastasis [54]. Selective inhibition of NF-κB and PAD4-dependent NETs could serve as an effective therapeutic strategy for breast cancer. Recent research has identified PAD4 as a promising target for tumor therapy, with some studies indicating that highly tumor-targeted PAD4 inhibitors, modified with phenylbutyric acid (PBA), can inhibit tumors in vivo by specifically disrupting the PAD4-H3cit-NETs pathway in neutrophils [55].

#### γδ T Cells and IL-17A: a dual influence on neutrophil behavior in breast cancer

Γδ T cells, a subtype of innate-like T lymphocytes, play a dual role in the development of breast cancer within the TME. They can secrete interferon (IFN)-γ to directly kill tumor cells, but they also produce interleukin-17A (IL-17A), which promotes tumor growth [56]. Angiogenesis, a critical physiological process and hallmark of cancer, is commonly targeted by antiangiogenic therapies, primarily those directed against the VEGF/VEGFR2 signaling axis. However, such treatments are limited in their efficacy for breast cancer patients, as high-dose anti-VEGFR2 therapy can lead to drug resistance. Research has shown that γδ T cells and neutrophils are actively involved in resistance to high-dose anti-VEGFR2 therapy in mouse models of breast cancer. Notably, high-dose VEGFR2-tyrosine kinase inhibitors have been found to induce γδ T cells to produce IL-17A via activation of the VEGFR1-PI3K-AKT pathway, which subsequently promotes N2-like neutrophil polarization. N2 neutrophils accelerate CD8^+^ T-cell exhaustion and foster an immunosuppressive microenvironment, thereby facilitating tumor progression [57].

#### The role of m6A demethylation in breast cancer neutrophils

Senescent neutrophils secrete exosomal piR-17560 in a STAT3-dependent manner, which enhances the expression of fat mass and obesity-associated protein (FTO) in breast cancer. The upregulation of FTO stabilizes and increases the expression of ZEB1, a key transcription factor that initiates EMT in cancer cells, by reducing N6-methyladenosine (m6A) RNA methylation. This alteration contributes to chemoresistance and EMT in tumor cells [58]. Additionally, a novel subset of C5aR1-positive neutrophils has been identified to secrete IL-1β and TNFα, which cooperatively activate extracellular signal-regulated kinase 1/2 (ERK1/2) signaling pathways. This activation leads to the phosphorylation of Wilms' tumor 1-associating protein (WTAP) at serine 341, thereby stabilizing the WTAP protein. The stabilization of WTAP further promotes the m6A methylation of enolase 1 (ENO1), influencing the glycolytic activity of breast cancer cells and thereby promoting tumor growth in vivo; this effect is abolished following WTAP silencing [59].

### Neutrophils as drivers of gastric cancer progression

Gastric cancer is a leading cause of cancer-related deaths, particularly in low- and middle-income countries. Despite advancements in research, the pathogenesis of gastric cancer remains poorly understood. The presence of neutrophils in tumors has been recognized as an independent and adverse prognostic factor for gastric cancer patients, with studies indicating that an elevated NLR is associated with poor survival outcomes [60]. This section summarizes several pathways through which neutrophils contribute to the progression of gastric cancer.

#### The GM-CSF-PD-L1 axis in gastric cancer neutrophil regulation

In human gastric cancer tissue samples, it has been observed that gastric cancer cells extend the lifespan of neutrophils, with tumor-derived granulocyte–macrophage colony-stimulating factor (GM-CSF) effectively activating neutrophils and inducing PD-L1 expression via the Janus kinase (JAK)-signal transducer and activator of transcription 3 (STAT3) signaling pathway. These PD-L1-expressing neutrophils subsequently suppress T-cell function, contributing to the evasion of anti-tumor immunity and promoting gastric cancer progression. Additionally, gastric cancer cell-derived extracellular vesicles (GC-EVs) have been shown to transport high-mobility group box-1 (HMGB1), which activates STAT3 and upregulates PD-L1 expression in neutrophils. Blocking the STAT3 pathway and silencing HMGB1 reverse this GC-EV-induced PD-L1 expression in neutrophils [61]. The prognostic value of the gastric immune prognostic index (GIPI) in patients treated with PD-1/PD-L1 inhibitors is also a growing area of interest.

#### Neutrophils on EMT in gastric cancer

EMT is a well-characterized process that plays a crucial role in cancer metastasis. In gastric cancer tissues, neutrophils produce IL-17A, and TANs-derived IL-17A promotes the migration, invasiveness, and EMT of gastric cancer cells by activating the JAK2/STAT3 pathway. Inhibition of this pathway, using IL-17A neutralizing antibodies or the JAK2-specific inhibitor AG490, reverses these TAN-induced phenotypes in gastric cancer cells [62]. Furthermore, research has shown that tumor cells activate neutrophils via TGF-β1, leading to the production of the metabolism-regulating signaling molecule FAM3C through Smad2/3 signaling. FAM3C, in turn, promotes EMT in tumor cells via the JNK-ZEB1/Snail signaling pathway [63]. Additionally, the deregulated expression of chemokines, such as CXCL5, in the TME contributes to tumor metastasis by inducing EMT and mediating the pro-tumor activation of neutrophils [64].

#### The IL-6/STAT3 axis in neutrophil-mediated gastric cancer progression

Cancer-associated fibroblasts (CAFs) are key players in cancer progression, largely through the secretion of growth factors, inflammatory extracellular matrix proteins, and proteases. Mesenchymal stem cells (MSCs), which possess self-renewal and pluripotent differentiation abilities, interact intensively with tumor-infiltrating neutrophils. Studies have shown that gastric cancer-derived MSCs activate neutrophils via the IL-6/STAT3 axis, promoting the migration of gastric cancer cells and the formation of endothelial cell tubes in vitro. The activated neutrophils, in turn, induce the differentiation of normal MSCs into CAFs [25].

#### The role of NETs and COX-2 in gastric cancer metastasis

NETs are critical in the progression of various cancers. Cyclooxygenase-2 (COX-2) has emerged as a potential target for gastric cancer immunotherapy. In vitro experiments have demonstrated that NETs can activate COX-2 through toll-like receptor 2 (TLR2), thereby enhancing the metastatic potential of gastric cancer cells. Additionally, a liver metastasis model in nude mice has highlighted the pivotal role of NETs and COX-2 in the distant metastasis of gastric cancer [65].

### Neutrophil dynamics in CRC

CRC is among the most common causes of cancer-related deaths worldwide, with an increasing number of cases reported each year [66]. The majority of CRC-related deaths are due to distant metastases, particularly liver metastases. Similar to other cancers, a high neutrophil-to-lymphocyte ratio (NLR) has been associated with poor clinical outcomes in CRC patients [67]. TANs exhibit plasticity between an anti-tumoral N1 phenotype and a pro-tumoral N2 phenotype, depending on signals from the surrounding tissue, such as TGF-β. Growing evidence suggests that neutrophils play a dual role in CRC development. Patients with CRC can exhibit elevated levels of NETs both in vivo and in vitro [68], with these NETs primarily localized within the primary tumor sites and at the tumor margins. High IL-8 expression in the CRC microenvironment activates neutrophils and induces NET formation. IL-8, through its receptor CXCR2, stimulates neutrophils to release NETs via Src, ERK, and p38 signaling, with these NETs subsequently upregulating TLR9 pathways to promote cancer progression [69]. A recent mouse study demonstrated that surgical trauma enhances colon cancer cell adhesion and growth on the peritoneal wall via CXCR2 signaling [70]. Moreover, NET-associated carcinoembryonic antigen-related cell adhesion molecule 1 (CEACAM1) has been shown to facilitate the relocation of CRC cells to the liver, both in vitro and in vivo [71]. In a mouse model of liver metastasis and surgical stress, inhibition of NET formation with DNase I reduced postoperative metastasis development. This accumulating evidence positions NETs as a potential therapeutic target for CRC.

Recent studies have also identified that KIAA1199 activates the TGF-β signaling pathway by interacting with TGFBR1/2, stimulating the production of CXCL1 and CXCL3, which facilitate the infiltration of immunosuppressive neutrophils into the liver [72]. Additionally, anterior gradient 2 (AGR2), a protein widely expressed in tumors, including precancerous lesions, primary tumors, and metastatic sites, is increasingly recognized for its role in promoting CRC metastasis [73]. Research has identified TANs as the predominant source of AGR2 in the TME, and TANs have been shown to drive CRC metastasis via the AGR2-CD98hc-xCT axis. Furthermore, in mouse models, the long non-coding RNA MIR4435-2HG (LINC00978) has been found to exert anti-tumor effects by reprogramming neutrophils [74].

## The role of neutrophils in different cancer types

Neutrophils exhibit context-dependent roles across various cancer types, demonstrating both pro-tumorigenic and anti-tumorigenic functions shaped by the TME. In HCC, neutrophils contribute to tumor progression through IL-6 and TNF-α-mediated inflammation, promoting angiogenesis and immune suppression via mechanisms involving arginase-1 and ROS [78]. In contrast, breast cancer neutrophils are known to facilitate metastasis primarily through the formation of NETs, which remodel the extracellular matrix (ECM) and can also display direct tumoricidal activity, demonstrating a more aggressive role [79]. Meanwhile, in gastric cancer, neutrophils are associated with poor prognosis, largely fostering immune evasion through matrix metalloproteinase-driven ECM degradation and the secretion of immunosuppressive cytokines [80]. In CRC, neutrophils exhibit a dual role: in early-stage tumors or under specific therapies, they may trigger cytotoxic effects against tumor cells, while in advanced stages, they are more likely to employ ROS and arginase-1 to suppress T-cell activity and promote a pro-metastatic niche. Despite these differences, certain mechanisms are conserved across cancer types [81]. For example, NETs and PD-L1-mediated immune checkpoint inhibition are common pathways through which neutrophils promote metastasis and immune evasion. Additionally, cancer type-specific metabolic adaptations, such as Acod1 expression in breast cancer versus ARG1 in CRC, illustrate how neutrophils can vary in their functional roles based on the tumor context. Microenvironmental drivers also play a significant role in shaping neutrophil functions; for instance, IL-6 is prominent in HCC, while CXCR2 signaling is vital in glioma. These factors underscore the functional plasticity of neutrophils in distinct tumor settings [82]. Ultimately, while neutrophils share mechanisms like ECM remodeling and immune modulation across cancers, their functional plasticity—heavily influenced by TME heterogeneity, disease stage, and therapeutic interventions—highlights their potential as dynamic therapeutic targets or prognostic biomarkers in cancer.

## The anti-tumor function of neutrophils

As previously discussed, TGF-β can induce neutrophil differentiation into a pro-tumoral phenotype (N2). By inhibiting TGF-β signaling, TANs can differentiate into an anti-tumor phenotype, characterized by elevated levels of pro-inflammatory cytokines and enhanced cytotoxicity, which induces tumor cell apoptosis and delays tumor growth in vivo [6]. TGF-β is a critical regulator of TANs plasticity, driving their differentiation into a pro-tumoral N2 phenotype characterized by immunosuppressive functions and tissue-remodeling properties. Mechanistically, TGF-β signaling via Smad2/3 phosphorylation suppresses pro-inflammatory pathways while upregulating arginase-1 and MMPs, facilitating tumor angiogenesis and extracellular matrix degradation, thereby promoting metastasis [83]. Inhibition of TGF-β signaling—through genetic knockout of TGF-β receptors or pharmacological agents like galunisertib—shifts TANs toward an anti-tumor N1 phenotype. This transition is marked by increased expression of pro-inflammatory cytokines (e.g., TNF-α, IL-12) and enhanced ROS-mediated cytotoxicity, leading to tumor cell apoptosis and delayed tumor progression in murine models [84].

Low-dose type I interferon (IFN-α) therapy has emerged as a potent inducer of the N1 phenotype. In HCC models, IFN-α stimulates neutrophil STAT1/IRF signaling, upregulating surface markers like ICAM-1 and FAS (factor-related apoptosis), which enhance neutrophil-tumor cell adhesion and direct cytotoxicity [85]. IFN-α-primed N1 neutrophils exhibit heightened production of myeloperoxidase and hydrogen peroxide (H_2_O_2_), which induce DNA damage in tumor cells via oxidative stress pathways [86]. Additionally, IFN-α enhances NETs formation, which can directly entrap circulating tumor cells and limit metastatic dissemination in breast cancer models [87].

Anti-tumor neutrophils exert direct cytotoxic effects on tumor cells while also activating adaptive immune responses indirectly. N1 neutrophils have demonstrated anti-tumor efficacy across various tumor models, including hepatocellular carcinoma and breast cancer. For instance, N1 neutrophils can directly kill tumor cells, limit their metastasis, and engage innate immune functions through the secretion of myeloperoxidase (MPO), H_2_O_2_, and ROS [88]. Furthermore, N1 neutrophils recruit dendritic cells (DCs), CD8^+^ T cells, CD4^+^ T cells, and other immune cells, promoting their proliferation and activation while regulating adaptive immunity through the release of cytokines and chemokines such as IL-8, growth-related oncogene (GRO)-α, macrophage inflammatory protein (MIP)-1α, MIP-1β, IL-12, tumor necrosis factor-alpha (TNF-α), CXCL10, and granulocyte–macrophage colony-stimulating factor (GM-CSF). One of the primary mechanisms by which N1 TANs exert their anti-tumoral effects is by enhancing the activity of cytotoxic T lymphocytes (CTLs). N1 TANs secrete chemokines and cytokines that recruit and activate CTLs, increasing their infiltration into the tumor site and enhancing their ability to target and kill tumor cells. This interaction is crucial, as CTLs are key effector cells in anti-tumor immunity, capable of inducing apoptosis in cancer cells through the release of perforin and granzymes [89]. Additionally, N1 TANs can modulate the activity of other innate immune cells, such as macrophages. By influencing the polarization of macrophages toward the M1 phenotype, which is known for its pro-inflammatory and anti-tumorigenic properties, N1 TANs contribute to the overall anti-tumoral milieu within the TME. This polarization is characterized by the production of pro-inflammatory cytokines and enhanced phagocytic activity, collectively working to suppress tumor growth and metastasis [90]. Furthermore, the interaction between N1 TANs and regulatory T cells (Tregs) is noteworthy. N1 TANs can inhibit the suppressive functions of Tregs, thereby alleviating the immunosuppressive pressure within the TME. This inhibition allows for a more effective immune response by preventing Tregs from dampening the activity of effector T cells and other immune components critical for tumor suppression [91].

In summary, N1 TANs play a multifaceted role in tumor suppression by interacting with and modulating the activity of various immune cells within the TME. These interactions enhance the anti-tumoral immune response, making N1 TANs a critical component of the anti-cancer immune system. A deeper understanding of these interactions could lead to the development of novel therapeutic strategies that harness the potential of N1 TANs in cancer immunotherapy.

## The dual roles of NETs across cancer types

NETs play significant roles in cancer biology, exhibiting dual roles that can either promote or inhibit cancer progression. These web-like structures, composed of DNA, histones, and various proteins, are released by neutrophils in response to various stimuli, including cancer cells. Their dual roles in cancer are multifaceted and context-dependent, influencing tumor progression, metastasis, and the TME.

On one hand, NETs can aid cancer progression by promoting tumor cell proliferation, invasion, and metastasis. They facilitate extracellular matrix remodeling, enhance angiogenesis, and create a pro-inflammatory environment that supports tumor growth. NETs trap circulating tumor cells, assisting in their dissemination and establishment at distant sites. Additionally, they can induce EMT in cancer cells, a key process in metastasis, by releasing factors that activate signaling pathways involved in EMT [92].

Conversely, NETs also contribute to anti-tumor immunity by trapping and killing cancer cells through the release of cytotoxic proteins and enzymes. They can interact with other immune cells, such as dendritic cells and macrophages, potentially enhancing the anti-tumor immune response. However, the balance between their pro-tumor and anti-tumor effects is delicate and influenced by various factors, including the type of cancer, the stage of disease, and the specific components of the TME [93].

The dual roles of NETs highlight their potential as therapeutic targets. Modulating NET formation or function could provide novel cancer therapies. For instance, inhibiting NET formation might reduce metastasis, while enhancing their anti-tumor effects could boost immune-mediated tumor clearance. Understanding how NETs influence cancer biology is crucial for developing targeted therapies that can exploit their dual roles to improve cancer treatment outcomes [94].

## Targeting tumor-associated neutrophils for cancer therapy

One promising therapeutic strategy involves inhibiting the recruitment of TANs and their downstream signaling pathways using small molecule inhibitors or neutralizing antibodies to impede tumor progression (Table 2). Various monoclonal antibodies (mAbs), small molecule inhibitors, and RNA interference techniques have been employed to block the signaling pathways between TANs and tumors. For example, the accumulation of N2 TANs can be reduced through the use of IL-8 antagonists, significantly delaying tumor growth in murine models [95]. TANs secrete IL-17A via the JAK2/STAT3 signaling pathway, which promotes EMT in gastric cancer cells. Inhibition of IL-17A signaling using polyclonal neutralizing antibodies or targeting the JAK2/STAT3 pathway with the inhibitor AG490 has been shown to reduce TAN-mediated migration and invasion [62]. Additionally, a study found that the tyrosine kinase inhibitor cabozantinib triggers a neutrophil-mediated anti-cancer innate immune response, facilitating tumor clearance [96]. Inhibition of CXCR1/2 can also block the recruitment of immunosuppressive neutrophils, thereby enhancing tumor response to PD-1 therapy [97]. Notably, the injection of β-dextran into normal mice has been found to reprogram TANs to the N1 phenotype, enabling them to suppress tumor growth independently of adaptation, with this tumor-suppressive capability maintained after transplantation into recipient mice [98]. Future research should focus on accurately targeting the clearance of N2 TANs while preserving N1 TANs.

**Table 2:** Some drugs related to TANs

Targets	Drug	Cancer application	Mechanism	Alone or in combination
TGF-β	LY2109761	Gastric cancer	Inhibit N2 neutrophils polarization and enhance tumor suppression capacity	LY2109761 + Oxaliplatin
CXCR2	Reparixin	Breast cancer	Diminish focal adhesion kinase (FAK) phosphorylation	Alone
	AZD5069	Prostatic cancer	Inhibit cancer cell survival through the inactivation of AR, HIF-1 and NF-Ƙb	AZD5069 + enzaluztamide
CXCR4 SIRPα CD47 TRAIL	BL-8040 KWAR23 Hu5F9-G4 Drozitumab	Pancreatic cancer Burkitt’s lymphoma Ovarian cancer Colorectal cancer	Exhibit a robust affinity for CXCR4 Bind human SIRPα with high affinity and disrupts its binding to CD47 Inhibit the SIRPα/CD47 pathway to enhance the tumor infiltrating Activate the TRAIL receptor to kill tumor cells	Alone Alone Alone Alone

*AR* androgen receptor, *HIF* hypoxia-inducible factor, *NF* nuclear factor

In summary, the diversity of transcription factors and specific proteins contributes to the variable phenotypes of TANs. Among the most extensively studied populations are N1 and N2 neutrophils. IFN-α is a key factor in polarizing neutrophils to the N1 phenotype, enhancing their adhesion, transmigration, phagocytosis, oxidative bursts, deregulation, and NET formation. Conversely, TGF-β drives neutrophil differentiation toward the N2 phenotype, which plays a modulatory role in immune responses. This switch between N1 and N2 phenotypes suggests an antagonistic relationship between IFN-α and TGF-β signaling pathways [84]. Precisely targeting N2 TANs without disrupting the normal immune function of patients with tumors remains a critical issue.

To date, several preclinical and clinical studies have evaluated the efficacy and safety of neutrophil-targeted or -related cancer therapies. For instance, one innovative study explored the combination of peroxynitrite (ONOO–)-mediated radio-sensitization with the polarization of TANs to reverse the immunosuppressive TME. This approach significantly amplified the effectiveness of radiotherapy in orthotopic mouse models of CRC and melanoma [99]. In other research involving mouse models, RCT001, a specific CXCR2 inhibitor, was shown to enhance the efficacy of combined anti-CTLA4 and anti-PD-1 therapies by inhibiting tumor-associated M2 macrophages and TANs [100]. Additionally, Deltex E3 ubiquitin ligase 2 (DTX2) in HCC cells has been identified as a promoter of TAN infiltration and polarization toward a pro-tumor phenotype. In vivo studies using various tumor models demonstrated that treatment with a DTX2 inhibitor not only inhibited tumor growth but also sensitized HCC cells to the therapeutic effects of PD-1 antibodies [101].

Notably, immunotherapies targeting neutrophils can result in unavoidable side effects. Agents such as DS-8273a, capecitabine, and gemcitabine are associated with side effects, including non-selective neutrophil depletion [102]. While selective removal of neutrophils presents a potential therapeutic avenue, this approach may inadvertently eliminate other immune cells, such as monocytes and specific subsets of CD8^+^ T cells [103]. Specific therapies targeting TANs and circulating neutrophils represent a novel approach for cancer treatment. Nevertheless, further investigation into the precise roles, recruitment pathways, subpopulations, and mechanisms of action of TANs is essential for developing a more reliable and specific treatment model that maximizes therapeutic potential while minimizing adverse effects.

## Conclusions and future research

This review sought to elucidate the intricate interactions between TANs and tumor cells, with a particular emphasis on the regulatory role of neutrophils in both promoting and combating cancer (Fig. 2 ). Neutrophils represent a highly heterogeneous population, exhibiting significant phenotypic and functional plasticity. In the TME, the majority of TANs contribute to tumor progression by promoting tumor cell proliferation, metastasis, angiogenesis, tissue remodeling, and immunosuppression. The infiltration of TANs is generally associated with poor prognosis across various malignancies. Numerous factors derived from the TME orchestrate the release, recruitment, and functional polarization of neutrophils. Conversely, neutrophil-secreted factors also play a crucial regulatory role in tumor growth. The effectiveness of tumor cell responses to immunotherapy is influenced not only by the mutation and reprogramming of their own genes but also by the complexity within the TME and the regulation of various cytokines and chemokines. A deeper understanding of the molecules through which TANs interact with the TME, along with the multiple signaling pathways involved, could lead to the reshaping of the TME into one that favors anti-tumor responses. This presents a promising new avenue for targeted tumor immunotherapy. As we move forward in understanding the complex roles of TANs in solid tumors, several key research directions should be prioritized. First, elucidating the molecular drivers behind N1/N2 polarization through advanced techniques such as single-cell multi-omics and spatial profiling will provide deeper insights into the regulatory mechanisms that guide neutrophil behavior within the TME. Additionally, defining the interactions between TANs and various immune and stromal cells is crucial for understanding their functional context. This includes resolving the dual roles of NETs in both metastasis promotion and suppression, as this knowledge may lead to innovative therapeutic strategies. Future research should also focus on metabolic reprogramming strategies for TANs, the development of engineered neutrophil carriers for targeted drug delivery, and the exploration of novel pathways, particularly the interplay between CXCR2 signaling and combinations of TGF-β and IFN-α. Addressing challenges such as clinical translation—including the identification of reliable biomarkers and understanding neutrophil lifespan in the tumor context—is essential for the successful implementation of neutrophil-targeted therapies. Moreover, investigating the influences of the microbiome on neutrophil function and its implications for cancer treatment will be a vital area of research. The integration of artificial intelligence (AI) in modeling TAN behavior could provide predictive insights and facilitate the design of more effective therapies. It is also important to consider the impacts of aging and comorbidities on neutrophil function and cancer progression, as these factors can significantly influence treatment outcomes. Optimizing selective NETosis inhibition could further enhance therapeutic efficacy while minimizing potential side effects. Finally, interdisciplinary approaches that combine immunology, oncology, and bioengineering, along with the development of advanced preclinical models, are critical for harnessing the potential of TANs in cancer immunotherapy. By pursuing these research directions, we can significantly advance our understanding of TANs and develop effective strategies to leverage their functions in treating solid tumors.

In conclusion, unraveling the regulatory pathways governing TAN-tumor interactions will be key to enhancing current immunotherapies by harnessing and manipulating these complex molecular mechanisms.

## Data Availability

No datasets were generated or analyzed during the current study.
